# Monitoring of Selected Swine Viral Diseases in Peruvian Amazon Peccaries

**DOI:** 10.1007/s10393-024-01692-9

**Published:** 2025-01-11

**Authors:** Maria F. Menajovsky, Pedro Mayor, Richard Bodmer, Pedro Pérez-Peña, Gabriela M. Ulloa, Alex D. Greenwood, Stephanie Montero, Andrés G. Lescano, Meddly L. Santolalla, Joaquim Segalés, Marina Sibila, Oscar Cabezón, Johan Espunyes

**Affiliations:** 1 Departament de Sanitat i Anatomia Animals, Facultat de Veterinària, Universitat Autònoma de Barcelona, Edifici V, Bellaterra, 08193 Barcelona, Spain; 2 ComFauna, Comunidad de Manejo de Fauna Silvestre en la Amazonía y en Latinoamérica, Iquitos, Peru; 3 Museo de Culturas Indígenas Amazónicas, Iquitos, Peru; 4 School of Anthropology and Conservation, Durrell Institute of Conservation and Ecology, University of Kent, Canterbury, UK; 5 Instituto de Investigaciones de la Amazonía Peruana (IIAP), Iquitos, Peru; 6 Emerge, Emerging Diseases and Climate Change Research Unit, School of Public Health and Administration, Universidad Peruana Cayetano Heredia, Lima, Peru; 7 Programa de Pós-Graduação em Saúde e Produção Animal na Amazônia, Universidade Federal Rural da Amazônia (UFRA), Pará, Brazil; 8 Grupo Enfermedades Emergentes, Universidad Científica del Sur, Lima, Peru; 9 Department of Wildlife Diseases, Leibniz Institute for Zoo and Wildlife Research, Berlin, Germany; 10 School of Veterinary Medicine, Freie Universität Berlin, Berlin, Germany; 11 Clima, Latin American Center of Excellence for Climate Change and Health, and Emerge, Emerging Diseases and Climate Change Research Unit, Universidad Peruana Cayetano Heredia, Lima, Peru; 12 Centre de Recerca en Sanitat Animal (CReSA), Unitat Mixta d’Investigació IRTA-UAB en Sanitat Animal, Campus de la UniversitatAutònoma de Barcelona (UAB), 08193 Bellaterra, Catalonia Spain; 13 WOAH Collaborating Centre for the Research and Control of Emerging and Re-Emerging Swine Diseases in Europe (IRTA-CreSA), 08193 Bellaterra, Spain; 14 IRTA. Programa de Sanitat Animal. Centre de Recerca en Sanitat Animal (CReSA), Campus de la Universitat Autònoma de Barcelona (UAB), 08193 Bellaterra, Spain; 15 Wildlife Conservation Medicine Research Group (WildCoM), Departament de Medicina i Cirurgia Animals, Universitat Autònoma de Barcelona, 08193 Bellaterra, Spain

**Keywords:** *Tayassu pecari*, *Pecari tajacu*, Epidemiology, Population dynamics, Classical swine fever, Aujeszky’s disease

## Abstract

Peccaries (collared peccary—CP—and white-lipped peccary—WLP) are an essential source of protein and income for rural communities in the Amazon region. Since 1980s, researchers in the Amazon have reported recurrent local disappearances of WLP populations. Although such disappearances impact the species conservation and the food security of rural societies, no studies have drawn consistent conclusions about the causes of these population collapses. However, it has recently been proposed that the overabundance of this species before its decline would be related to infectious disease outbreaks. In the current study, we aimed to determine the circulation (occurrence and exposure) of viruses relevant to swine health in CP and WLP populations, namely classical swine fever virus (CSFV), Aujeszky's disease virus (ADV), swine vesicular disease virus (SVDV), and porcine circoviruses (PCV). The study was conducted in two areas of the northeastern Peruvian Amazon: the Yavarí-Mirín River basin (2008 -2020), where WLPs experienced extreme population fluctuations, and the Pucacuro National Reserve (2012–2014), where no WLP disappearances have been reported. Since WLP is not easily found during population declines, we also sampled CP as an indicator of virus circulation in the area as they are likely to be susceptible to the same pathogens. CSFV and ADV antibodies were detected in both peccary species and both areas. Diseases caused by CSFV and ADV have the potential to act as ultimate causes of population collapse, especially in large WLP populations where overabundance could increase the rate of pathogen transmission. Our results were inconclusive in establishing whether or not these viruses drove the WLP population to collapse, but their potential role warrants deeper investigation, expanding the geographical coverage of studies on infectious diseases in peccaries.

## Introduction

Peccaries (Cetartiodactyla: Tayassuidae) are native to Latin America and are distributed from Mexico to northern Argentina (Sowls, 1997). They play a pivotal role in ecosystem dynamics by rooting soils, dispersing seeds and seedlings, consuming plant and animal matter, and serving as prey for top predators (e.g., *Panthera onca* and *Puma concolor*) (Sobral et al., 2017; Weckel et al., 2006; Porfirio et al., 2017). In the Amazon, two species of peccaries coexist: the white-lipped peccary (WLP, *Tayassu pecari*) and the collared peccary (CP, *Pecari tajacu*).

Subsistence hunting is essential to traditional livelihoods and an important source of protein and income for rural communities—including indigenous and campesino communities—and cities in the Amazon region (Bodmer & Pezo, 2001; Mayor et al., 2022). In the early 2000s, the annual harvest in the rural Peruvian and the Brazilian Amazon was estimated to be approximately 14,000 WLP and 20,000 CP individuals (Bodmer & Pezo, 2001), and 611,527 WLP and 551,949 CP individuals (Peres, 2000), respectively. In addition, peccaries are also of enormous socioeconomic importance as they are the most traded wild meat species in urban markets, accounting for 43 tons per year (17.9% of the total biomass traded) of WLP meat and 97 tons (39.8%) of CP meat in Iquitos, the largest urban market in the Peruvian Amazon (Mayor et al., 2022).

Since 1980s, researchers have reported recurrent local disappearances of WLP populations across their Latin American range, spanning at least 50 million hectares (Richard-Hansen et al., 2014; Fragoso et al., 2016; 2022), and affecting the ecosystem dynamics, as well as the food supply and economic activities of indigenous and rural communities (Fragoso et al., 2016; 2022). These population cycles appear to last 20–30 years, with rapid population decline occurring in the first 1–5 years, followed by the absence or low abundance for the next 7–12 years, and finally a slow growth over the next 20 years (Fragoso et al., 2022). However, not all populations recover. Currently, the WLP, now considered ‘Vulnerable’ by the *International Union for Conservation of Nature* (IUCN, 2023), is found in only 21% of its recognized distribution geographic range (Taber et al., 2021).

Early studies attributed these populations’ oscillations to migrations, overhunting, and environmental changes (such as land-use changes), but none have reached consistent conclusions (Altrichter et al., 2012; Fragoso et al., 2022). The density-dependent overcompensation theory has recently gained prominence, suggesting that WLP population overabundance triggers density-dependent effects, such as increased pathogen transmission, resulting in disease outbreaks and population decline (Fragoso et al., 2022). Even though both WLP and CP are influenced by the same environmental conditions, have similar reproduction cycles (Mayor et al., 2017), and are similar hunting preferences for subsistence hunters (Pérez-Peña et al., 2021), such disappearance cycles have not been reported for CP. However, there are several ecological differences between the two species. WLP weigh 30–50 kg and form herds of around 400 individuals with a home range of 16–200km^2^ (Fragoso, 1998; Kiltie & Terborgh, 1983), whereas CP weigh 25 kg on average and form herds of 3–20 individuals with a home range of 1–7km^2^ (Keuroghlian et al., 2004). In general, WLP occupy densities ranging from 3.7 to 25 individuals/km^2^, while CP occupy densities ranging from 2.8 to 9.8 individuals/km^2^ (Fragoso, 1998; Keuroghlian et al., 2004). These differences may influence how animals respond to pathogens and disease outbreaks (Fragoso et al., 2022).

Several swine viruses, including classical swine fever virus (CSFV), Aujeszky’s disease virus (ADV), and swine vesicular disease virus (SVDV), as well as various porcine circoviruses (PCV), have a significant impact on swine health, affecting the reproductive, nervous, respiratory, and gastrointestinal systems, with high associated mortality rates that can reach 100% (Postel et al., 2018; Zuckermann, 2000; WOAH, 2023). However, to our knowledge, there is a significant gap in information about the occurrence and health impact of infectious diseases on wildlife in the Amazon region. Indeed, diseased WLP and carcasses have been observed during periods of population decline in some regions, but further examination was not carried out (Fragoso et al., 2022). In the Southern Peruvian Amazon, studies have reported the occurrence of antibodies against ADV in free-living WLP populations (Romero Solorio, 2010). Also, ADV and porcine circovirus 2 (PCV-2) have been reported in CP and WLP populations in the Bolivian and Brazilian Amazon but ignoring their impact on their population dynamics (Karesh et al., 1998; De Castro et al., 2014). PCV-2 has also been documented among domestic pigs in the Southern Brazilian Amazon (Dutra et al., 2013). Despite these few studies, pathogens pertinent to swine health in the Amazon region remain largely underexplored (Menajovsky et al., 2023).

WLP populations can experience explosive population growth (Fang et al., 2008) and due to the risk and rate of infectious disease transmission is higher in dense populations (Tarwater & Martin, 2001), we hypothesized that highly virulent infectious diseases may be involved in the severe population declines of WLP in the Amazon. However, the logistic difficulties of studying wildlife diseases in such remote areas are a major challenge, as evidenced by a lack of information about infectious diseases and their role in the decline of wild species populations, such as WLP (Menajovsky et al., 2023).

The present study aimed to improve the knowledge about the circulation of selected infectious diseases in areas of WLP fluctuations by assessing the seroprevalence and presence of major swine pathogens in CP and WLP populations in the Peruvian Amazon.

## Materials and Methods

### Study Area

The study was carried out in two areas of the Peruvian Amazon: the Yavarí-Mirín River (YMR) basin and the Pucacuro National Reserve. The Yavarí-Mirín River basin (YMR; 04°19′53’’S; 71°57′33’’W) is a remote area on the Peru-Brazil border, composed of a diverse landscape that ranges from upland forests with nutrient-poor sandy soils to flooded forests with relatively nutrient-rich soils (Ter Steege et al., 2003). The only village in YMR is Nueva Esperanza, a Yagua indigenous community of around 300 people. The main human activities of this community are traditional small-scale agriculture, fishing, logging, and subsistence hunting (Mayor et al., 2015). There is no large-scale agriculture in the study area; therefore, the crops are subsistence-based. In the YMR basin, WLPs have shown extreme population fluctuations over the last 25 years, including a decline from a high of 15 ind./km^2^ in 2000 to 2 ind./km^2^ in 2004 and a complete disappearance between 2005 and 2015 (Fang et al., 2008; Bodmer et al., 2024).

The Pucacuro National Reserve (PNR), located on the border with Ecuador (2°26′53″S 75°20′29″W), is composed of high terrace forests, non-flooded habitat with dissected relief in a humid tropical forest. PNR has a game species management plan that includes CP, WLP, and *Cuniculus paca*. Due to their large population sizes and relatively high reproductive capacity, management groups hunt these species for consumption and commercialization to support local indigenous hunters' economies (SERNANP, 2014). There have been no reports of WLP or CP population declines in PNR (Perez Peña et al., 2016).

**Figure 1:**
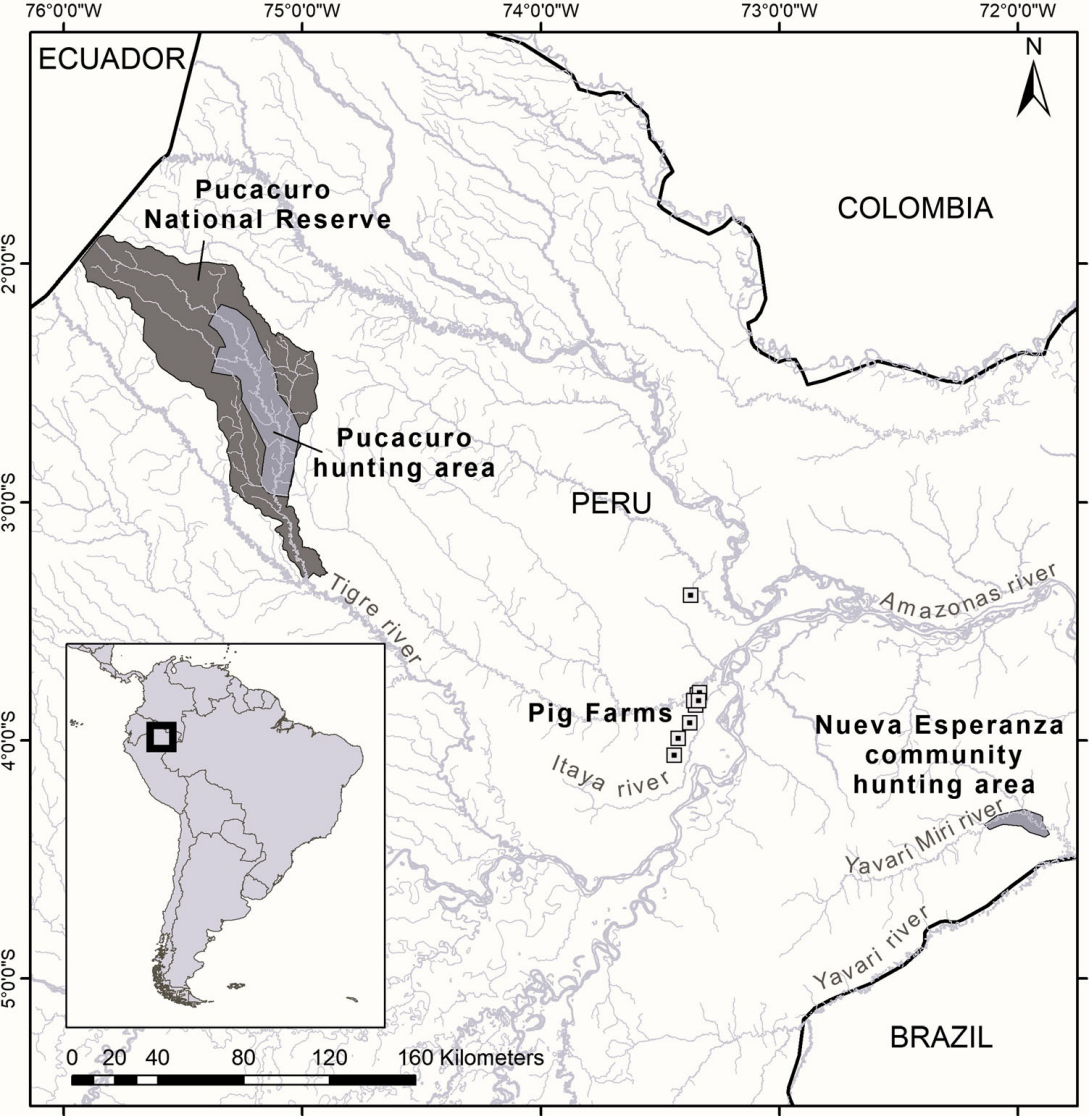
Map of the study area, including the hunting areas of Nueva Esperanza community in the Yavarí Mirin River basin and the Pucacuro National Reserve. The map also shows all pig farms registered in the region (squares; SENASA, pers. comm.).

Both study areas are highly biodiverse and well-preserved, with low human impact (Pitman et al., 2003; SERNANP, 2019), and the nearest pig farms are 160 and 170 km away, respectively, from YMR and PNR (Fig. 1).

### Biological Collection

Blood samples were obtained from both study areas, totaling samples from 98 WLP and 140 CP. The samples were collected by subsistence hunters and park rangers as part of a wildlife conservation program, taking advantage of the discarded material from legal subsistence hunting. Hunters impregnated blood from the cranial or caudal cava veins on either Whatman filter paper n. 3 or FTA® cards (Scheilcher & Schuell, Germany). The YMR basin was sampled from 2008 to 2015 and 2019 to 2020, while the PNR was sampled from 2012 to 2014. Samples were collected in all seasons, and hunters recorded the species, date, location, and sex. The samples were kept at room temperature in a sheltered environment in the study areas for 15 to 100 days before being stored at − 20°C until analyses (Aston et al., 2014; Menajovsky et al., 2024; Morales et al., 2017).

### Serological Analyses

A 132 mm^2^ area of the filter paper with blood was cut with a sterile punch (Staples®) and diluted in 400 µL of sterile PBS (phosphate-buffered saline). These samples were vortexed for 20 s, stored at 4°C for 24 h before being vortexed for 20 s, and frozen at − 20°C until analysis. As serum represents 40% of total blood volume (Nobuto, 1966) and the blood concentration on filter paper is 40 µL/cm^2^, we obtained a serum dilution of 1:20. The elutions were tested for antibodies against CSFV, ADV, and SVDV using the ID Screen Classical Swine Fever E2 Competition ELISA kit, the ID Screen Aujeszky gB Competition ELISA kit, and the ID Screen Swine Vesicular Disease Competition (IDvet, Montpellier, France), respectively.

### Molecular Analyses

RNA was extracted from 200 μL of the elutions using the commercial kit IndiMag® Pathogen Kit (Indical Bioscience GmbH, Leipzig, Germany) according to the manufacturer’s instructions. RNA concentrations were determined using the Qubit Fluorometric Quantification High Sensitivity Assay (Invitrogen, California, USA). Molecular detection of porcine circoviruses 1, 2, 3, and 4, as well as CSFV, was performed by conventional and RT-PCR, following previously described protocols (Vilček et al., 1994; Quintana et al., 2002; Oliver-Ferrando et al., 2016; Franzo et al., 2020; Saporiti et al., 2021). The 95% confidence intervals for antibodies and pathogen prevalence were calculated using the Wilson score method (Thrusfield, 2018).

### Statistical Analysis

We used Fisher’s test to compare seroprevalences of CSFV and ADV between all the animals analyzed in YMR and PNR, as well as between the two analyzed periods in YMR.

### Ethics

Field and laboratory procedures were previously authorized by Peruvian and International Authorities: Dirección General de Flora y Fauna Silvestre from Peru (041–2007- DGGFS-DGEFFS, 0350–2012-DGFFS-DGEFFS, 258–2019-MINAGRI-SERFOR-DGGSPFFS), the Head of the National Reserve of Pucacuro (03–2012-SERNANPRN Pucacuro), and the Institutional Animal Use Ethics Committee from the Universidad Peruana Cayetano Heredia (029–03-19, protocol #102,142) and the *Universitat Autònoma de Barcelona* (protocol #4829). Export permits were as follows: 003258-CITES-Perú, 003260-CITES-Perú, BB-00017 20I-Spain, and BB-00018 20I-Spain.

## Results

Six specimens presented antibodies against CSFV, three CP and three WLP, for an overall seroprevalence of 2.14% (CI_95%_: 0.70–6.11%) in CP and 3.06% (CI_95%_: 1.05–8.62) in WLP (Table 1). Seropositive CPs were only detected in YMR in 2009, 2013, and 2014, during the WLP population crash in the area. One seropositive WLP was detected in YMR in 2020 and two seropositive individuals in PNR in 2014. Antibodies against ADV were detected in 3.06% WLPs (3/98; CI_95%_: 1.05–8.62). Two seropositive individuals were hunted in YMR in 2019, during WLP population recovery, and one in PNR in 2014 (Table 1). All samples were negative for antibodies against SVDV. Molecular analyses for PCV-1, PCV-2, PCV-3, PCV-4, and CSFV were all negative.

**Table 1:** Results for the Detection of Antibodies Against Classical Swine Fever Virus (CSFV) and Aujeszky’s Disease Virus (ADV) in White-Lipped Peccary (WLP; *Tayassu pecari*) and Collared Peccary (CP; *Pecari tajacu*) in the Yavarí-Mirín River Basin and the Pucacuro National Reserve (Peruvian Amazon).

Area	Species	Period	CSFV	ADV
Yavarí-Mirín River basin	WLP	2008–2015	0/6 (0%) [0.00–39.03%]	0/6 (0%) [0.00–39.03%]
		2019–2020	1/49 (2.04%) [0.36–10.69%]	2/49 (4.08%) [1.13–13.71%]
		Total	1/55 (1.82%) [0.32–9.61]	2/55 (3.64%) [1.00–12.32%]
	CP	2008–2015	3/98 (3.06%) [1.05–8.62]	0/98 (0%) [0.00–3.77%]
		2019–2020	0/39 (0%) [0.00–8.97%]	0/39 (0%) [0.00–8.97%]
		Total	3/137 (2.20%) [0.75–6.24]	0/137 (0%) [0.00–2.73]
Pucacuro National Reserve	WLP	2012–2014	2/43 (4.65%) [1.28–15.46]	1/43 (2.33%) [0.41–12.06]
	CP	2012–2014	0/3 (0%) [0.00–56.15]	0/3 (0%) [0.00–56.15]

The 95% confidence intervals are presented in square brackets.

*CSFV* Classical swine fever virus, *ADV* Aujeszky’s disease virus.

Fisher tests did not reveal any significant difference in the seroprevalence between both areas or both periods.

## Discussion

Peccaries are essential to the food security of rural societies in the Amazon region (Bodmer & Pezo, 2001; Fragoso et al., 2022). Even though the extreme WLP population declines have led to its recategorization as “Vulnerable” by the IUCN (Keuroghlian et al., 2013), the impact of infectious diseases on these declines has not been assessed appropriately. Wildlife population declines are usually multifactorial processes, even if one cause can be identified as predominantly responsible (Sodhi et al., 2009). Many species are already threatened due to habitat fragmentation, declining genetic diversity, or overexploitation (Frank et al., 2006), and their combination with infectious diseases increases even more the risk of local extinctions (Smith et al. 2006).

Throughout the Amazon, at least 28 independent WLP disappearance events have been recorded (Fragoso et al., 2022). Since there is no published data on the infectious diseases’ impact in peccaries, and it is not clear which pathogens may be present in peccary populations, their assessment should be carefully addressed. In this sense, a recent study highlighted the necessity of performing longitudinal studies on infectious diseases in peccaries to address this gap of knowledge (Menajovsky et al., 2023).

The present study confirmed the exposure of Peruvian peccaries to CSFV and ADV. These viruses cause disease in domestic and feral pigs, being the latest ones considered as reservoir hosts for domestic pigs worldwide (Postel et al., 2018; Zuckermann, 2000). Classical swine fever (CSF) has not been previously reported in the Amazon region, but it is considered an endemic disease in domestic pigs from other regions in Brazil and Peru (Pereda et al., 2005; De Oliveira et al., 2020), and its circulation has also been reported in CP in non-Amazon areas in Colombia (Montenegro et al., 2018). Our results present evidence of peccaries contact with the virus in the Amazon region, as both WLP and CP were exposed to CSFV in both study areas. However, our results are inconclusive in establishing whether or not these viruses drove the WLP population to collapse. On the one hand, WLP were almost absent in the YMR basin between 2008 and 2015 (*n* = 6; 0% seroprevalence), when antibodies against CSFV were detected in collared peccaries (*n* = 98; 3.06% seroprevalence), tipping the balance in favor of the hypothesis of CSFV as a modulator of population dynamics of WLP. Antibodies against CSFV were only detected in WLP during the following years (2019–2020) when the population had recovered. On the other hand, antibodies against CSFV were detected in WLP from the PNR, where populations did not decline. Since CSFV is highly contagious and can cause large mortality outbreaks in domestic pigs and wild boars, a significant role of CSFV in the population declines of peccaries cannot be completely ruled out and warrants deeper investigation.

Aujeszky’s disease can also cause significant outbreaks in domestic pigs with high mortality and major economic consequences (Zuckermann, 2000). This suggests that the effect of the virus on the population dynamics of free-ranging wild boar appears could be more limited, probably due to the circulation of attenuated strains, as the experimental infection with virulent strains results in severe disease and death (Müller et al. 2001). As with CSFV, the susceptibility and impact of ADV on peccaries are still unknown but potentially significant in case of virulent strains. Seropositivity for antibodies against ADV has been previously observed in WLP from the Southern Peruvian and Bolivian Amazon, but not in CP (Romero Solorio, 2010; Karesh et al., 1998). In our study, antibodies were detected only in WLP and in both study areas, with similar low seroprevalences as in the previous studies. As with CSFV, antibodies were not detected in the YMR basin during the decline in the WLP population and were only detected in the following years. Similarly, these findings are inconclusive in establishing the role of ADV in the declines of WLP populations. However, they deserve further attention as the impact of the virus on peccaries is still unknown. In addition, ADV does not only affect swine species but can also cause significant mortality in carnivorous mammals, for example, in dogs and in wild species as *Puma concolor* (Zhang et al., 2015; Cunningham et al., 2021). Thus, the presence of ADV in peccaries may represent a conservation issue for both peccaries and endangered carnivore species.

We consider it unlikely that the pathogens studied were introduced through pig farms. Feral pigs have not been reported in either YMR or PNR, and pig farms are located near Iquitos, at least 160 km away from both sampling areas (Fig. 1). The maximum home range of CP is 7km^2^ and of WLP is 100–200km^2^ (Taber et al., 1994; Fragoso, 1998; Kiltie & Terborgh, 1983), and the presence of large rivers in the area as ecological barriers hinder contact with the aforementioned farms. Additionally, in front of our study, area lies the Brazilian territory, which includes Brazil’s largest Indigenous Reserve. These are areas where farming is prohibited (Verissimo et al., 2011). Consequently, we can reasonably infer that there is no spillover of pathogens from domestic pigs in the frontier area. However, the presence of traditional small-scale pig farming cannot be ruled out and should be further evaluated to understand the potential role of small-scale farming in the introduction of swine viruses in the free-living Amazon. In fact, it is possible that previous contacts with backyard pigs could have introduced these viruses within the free-living peccary population, where they have been naturally transmitted between and within their populations.

Antibodies against SVDV and genome from PCV-1, PCV-2, PCV-3, PCV-4, and CSFV were not detected in any sample from the study. Swine vesicular disease virus and the four porcine circoviruses are associated with different degrees of disease in pigs. From these, only PCV-2 has previously been reported in wild peccaries and domestic pigs in the Colombian Amazon (Montenegro et al., 2018) and the Brazilian Amazon and Pantanal (De Castro et al., 2014; Dutra et al., 2013). SVDV and PCV have not been reported in peccaries nor domestic pigs in the Amazon basin (Menajovsky et al., 2023), supporting our findings and evidencing their improbable role in the population dynamics of WLP.

Some technical constraints, such as sample conservation, must be considered when interpreting our results. Filter paper is a convenient technique when working with local communities in logistically challenging remote areas because it simplifies sample collection and storage, especially since an adequate cold chain is unavailable. Local communities rely on subsistence hunting for food and could become active samplers of valuable biological material that is often discarded. In previous studies, these same samples have already been used successfully to perform serological and molecular DNA analyses (Aston et al., 2014; Morales et al., 2017); however, it is important to consider that storage at room temperature under ambient conditions could potentially impact sample stability. This can lead to a higher likelihood of false negative results compared to analyzing frozen biological samples (Bevins et al., 2016), particularly when working with RNA viruses. Therefore, our results must be considered conservative, and the reported seroprevalence may be lower than reality. Furthermore, it is worth noting that PNR was sampled more than 10 years ago, and since then, the health status of the pecaries’ populations in the area might have changed. Additionally, we obtained few samples in the CP sampling in PNR and during the WLP population crash in YVR, these negative results may be underestimating circulating antibodies against CSFV, ADV or SVDV.

Determining infectious diseases’ impact on WLP disappearance episodes is challenging. The presence of a pathogen in a declining population does not imply that the pathogen played a significant role in the decline (McCallum, 2012). Baseline data still needs to be included to model the epidemiology and the impact of viruses like CSFV and ADV on peccaries (Menajovsky et al., 2023). Due to viral strains with different severity of disease (Pereda et al., 2005; De Oliveira et al., 2020), isolation and phylogenetic analysis of circulating strains in free-ranging peccaries and experimental infections are required to evaluate and understand their pathogenesis and potential effects. Expanding the geographical coverage of studies on infectious diseases in peccaries to include other areas where population declines have been reported would assist in determining whether pathogens are associated to population declines. We focused our study on viruses known to have a significant impact on swine health, but bacterial, parasitic, and other viral infections may also have repercussions on populations dynamics and should be questioned further. Because the fluctuations of WLP populations are likely to be multifactorial, it is also essential to include strategies for monitoring population dynamics that allow anticipating the population decline to develop surveillance before and during a WLP disappearance event.
